# Infantile Convulsions with Paroxysmal Dyskinesia (ICCA Syndrome) and Copy Number Variation at Human Chromosome 16p11

**DOI:** 10.1371/journal.pone.0013750

**Published:** 2010-10-29

**Authors:** Patrice Roll, Damien Sanlaville, Jennifer Cillario, Audrey Labalme, Nadine Bruneau, Annick Massacrier, Marc Délepine, Philippe Dessen, Vladimir Lazar, Andrée Robaglia-Schlupp, Gaëtan Lesca, Elisabeth Jouve, Gabrielle Rudolf, Jacques Rochette, G. Mark Lathrop, Pierre Szepetowski

**Affiliations:** 1 INSERM Unité 910, Marseille, France; 2 Université de la Méditerranée, Aix-Marseille, France; 3 Service de Cytogénétique Constitutionnelle, Hospices Civils de Lyon, Lyon, France; 4 EA 4171, Université Claude Bernard Lyon, Lyon, France; 5 INSERM Unité 901, Marseille, France; 6 Institut de Neurobiologie de la Méditerranée (INMED), Marseille, France; 7 Centre National de Génotypage (CNG), Université d'Evry, Evry, France; 8 Unité de Génomique Fonctionnelle, Institut de Cancérologie Gustave Roussy (IGR), Villejuif, France; 9 CIC-UPCET Unité de Pharmacologie Clinique et d'Evaluations Thérapeutiques, AP_HM, Marseille, France; 10 Clinique Neurologique, Hôpitaux Universitaires de Strasbourg, Strasbourg, France; 11 INSERM UMR925 & Unité de Neuro-Pédiatrie, Université de Picardie Jules Verne, Amiens, France; University Hospital Vall d'Hebron, Spain

## Abstract

**Background:**

Benign infantile convulsions and paroxysmal dyskinesia are episodic cerebral disorders that can share common genetic bases. They can be co-inherited as one single autosomal dominant trait (ICCA syndrome); the disease ICCA gene maps at chromosome 16p12-q12. Despite intensive and conventional mutation screening, the ICCA gene remains unknown to date. The critical area displays highly complicated genomic architecture and is the site of deletions and duplications associated with various diseases. The possibility that the ICCA syndrome is related to the existence of large-scale genomic alterations was addressed in the present study.

**Methodology/Principal Findings:**

A combination of whole genome and dedicated oligonucleotide array comparative genomic hybridization coupled with quantitative polymerase chain reaction was used. Low copy number of a region corresponding to a genomic variant (Variation_7105) located at 16p11 nearby the centromere was detected with statistical significance at much higher frequency in patients from ICCA families than in ethnically matched controls. The genomic variant showed no apparent difference in size and copy number between patients and controls, making it very unlikely that the genomic alteration detected here is ICCA-specific. Furthermore, no other genomic alteration that would directly cause the ICCA syndrome in those nine families was detected in the ICCA critical area.

**Conclusions/Significance:**

Our data excluded that inherited genomic deletion or duplication events directly cause the ICCA syndrome; rather, they help narrowing down the critical ICCA region dramatically and indicate that the disease ICCA genetic defect lies very close to or within Variation_7105 and hence should now be searched in the corresponding genomic area and its surrounding regions.

## Introduction

Benign familial infantile seizures (BFIS) (MIM 601764, 605751, 607745, 612627) correspond to non-febrile and focal convulsions with onset at age 3–12 months and a favorable outcome. Although their relative prevalence is not known with precision, they may be one of the most common epilepsy in the two first years of life[1]. BFIS is an autosomal dominant disorder with incomplete penetrance. Benign infantile seizures (BIS) may also be associated with paroxysmal dyskinesia (PD) in the context of the autosomal dominant ICCA (Infantile Convulsions with paroxysmal ChoreoAthetosis) syndrome (MIM 602066)[2]–[5]. PD represent a rare group of neurological movement disorders characterized by episodes of involuntary abnormal movements (dyskinesia) that correspond to attacks of choreoathetosis or dystonia. PD are heterogeneous at both the clinical and genetic levels, with several genes mapped and a subset identified at chromosomes 1p34 (*SLC2A1* gene) and 2q35 (*MR-1* gene)[6]–[8]. The epileptic origin of PD has long been a matter of debate[4], [5] and PD have even been classified as reflex epilepsies. Indeed, attacks of PD and epileptic seizures have several characteristics in common: both are paroxysmal in nature with a tendency to spontaneous remission, and a subset of PD responds well to anticonvulsants.

In 1997, description of the familial ICCA syndrome in four families from Northern of France and linkage to chromosome 16p12-q12 provided the first genetic evidence for common mechanisms shared by BFIS and by PD[2]. Since then, numerous studies have confirmed linkage of ICCA pedigrees[3], [9]–[11] to the same ICCA genomic area, strongly suggesting genetic homogeneity of the ICCA syndrome. Indeed, no ICCA family has ever been excluded for linkage to the ICCA genomic area amongst the 27 families that have been genotyped so far[2], [3], [9]–[14]. Noteworthingly, a majority of pure BFIS families[14]–[17] also showed linkage to the same ICCA genomic area. This suggested that the BFIS and ICCA syndromes that are linked to 16p12-q12 may be allelic. However, there may be several homologous disease genes in the ICCA region, where nearly identical copies of genes have been described[5]. If there were more than one disease gene in the ICCA region, determining a single and consensus critical region could lead to dramatic errors – notwithstanding artifacts of wrong genome assembly. To date, no such disease gene has been identified yet despite quite intensive conventional mutation screening (Roll, Massacrier, Délepine, Lathrop & Szepetowski, our unpublished data). The pericentromeric ICCA area displays highly duplicated large DNA sequences[18], [19] and this may present an enormous challenge for mutation search. Furthermore, the occurrence of large-scale genomic rearrangements such as deletions and duplications would be favored in the context of such a complicated genomic architecture and hence should be considered in physiological as well as in pathological conditions. Indeed, copy number variations of various sizes and locations have been reported in the recent years at human chromosome 16p12-q12 in normal individuals[19]–[23] (database of genomic variants at http://projects.tcag.ca/variation/?source=hg18), and deletion/duplications at 16p11.2 have been associated with higher risk for developmental disabilities[24], for autism spectrum disorders[25], [26], for severe obesity[27], [28], for schizophrenia[29] and for various other phenotypes such as speech, language and cognitive impairments, behavior problems or epileptic seizures[30]. Generally, an increasing number of human pathologies including nervous system diseases, have proved to be genomic disorders arising either *de novo* or as inherited traits[31], [32]. In the present study and using a combination of whole genome and dedicated oligonucleotide array comparative genomic hybridization (CGH) coupled with quantitative polymerase chain reaction (qPCR), we have explored the possibility that the ICCA syndrome is associated with genomic alterations in the pericentromeric region of human chromosome 16.

## Materials and Methods

### Ethics Statement

All experiments were conducted in accordance with the Declaration of Helsinki and all procedures were carried out with the adequate understanding and written consent of the subjects, and after approval from the appropriate ethical committees (‘Comité Consultatif de Protection des Personnes dans la Recherche Biomédicale de Marseille II’, St Paul Centre Hospital, Marseille; ‘Comité Consultatif de Protection des Personnes dans la Recherche Biomédicale d'Alsace n°1′ and ‘Comité de Protection des Personnes “EST IV”’, Strasbourg University Hospital).

### Patients and controls

All ICCA patients and families studied here have already been reported[2], [3], [13], [33]. The terms ‘ICCA patient’ and ‘ICCA family’refer to the ICCA syndrome, which is characterized by a variable presentation of BIS and PD in the same patient, or in different patients in one given family. Hence all individuals in a given ICCA family do have the ICCA syndrome inherited as one single autosomal dominant trait (and hence one single genetic defect is inherited), whether they present with BIS, with PD, or with both associated. DNAs from patients and from heatlhy controls were extracted, purified and validated (NanoDrop™ 1000, Thermo Fisher Scientific) from fresh blood samples according to standard procedures.

### CGH-array (aCGH) analyzes

A first set of experiments was performed using a 244 K Human CGH (G4411B) microarray (Agilent Technologies, Santa Clara, CA, USA). In all experiments, opposite sex-matched DNA from a pooled human female or male individual (Promega, Madison, WI) was used as the reference. Oligonucleotide aCGH processing was performed as detailed in the manufacturer's protocol (CGH-v4_91; http://www.agilent.com). Data were extracted from scanned images using an Agilent Scanner G2505B and feature extraction software (version A. 9.1.1.1, Agilent). Raw data text Files from the latter were then imported for analysis into CGH Analytics 3.4.40. Aberrations were detected with the ADM2 algorithm and filtering options of a minimum of five probes and abs(log2Ratio) 0.3. Aberration segments were individually reviewed using build 35, hg17 of UCSC.

A second series of experiments was performed using a 44,000 oligonucleotides Agilent Technologies® custom microarray, enriched in the region 16p12.1q12.1 between nucleotide (nt) positions 27,596,583 and 45,502,600. Design of the custom microarray was done at Imaxio (http://www.avidis.fr/index.php) and 12,305 oligonucleotides were added in this specific region, allowing an average resolution of 642 bp between two consecutive oligonucleotides (centromeric region was excluded). Eight hundred nanograms of patient's DNA or of reference DNA (Human Genomic DNA, Promega®, Madison) were digested respectively with *AluI* and *RsaI* (Promega®, Madison). Each digestion product was labeled by random priming with Cyanine 5 for patients or Cyanine 3 for the reference according to the Genomic DNA Labeling Kit protocol (Agilent®, Santa Clara). After columns-purification with Microcon (Millipore®, Bedford), each probe was denatured and pre-annealed with 50 µg of human Cot-1 DNA (Invitrogen®, California). Hybridization was performed at 65°C for 24 hours. After washing, array was analyzed by Feature Extraction® 9.5 software. Interpretation of the results was carried out with the CGH Analytics® 3.5.14 software, set with the following parameters: ADM-2, threshold: 6.0, window: 0.5 Mb, cut-off: 0.25.

### Quantitative PCR (qPCR) experiments

PCR primers were designed for two specific subregions (ICCA.SRa and ICCA.SRb, respectively) situated within Variation_7105, as well as for the 10p14 *GATA3* (GATA binding protein 3) control gene, using the Universal Probe Library Assay Design Center at https://www.roche-applied-science.com/. No copy-number change has been described in the human genome (Database of Genomic variants at http://projects.tcag.ca/variation/) for control *GATA3* amplicon and no genomic rearrangement was detected in the *GATA3* genomic area in the first series of whole genome CGH experiments. Primers were: for ICCA.SRa, forward 5′-caagtgagttcttgaaacatcca, and reverse 5′-tgaaatccatccctgctgat; for ICCA.SRb, forward 5′-ggttatctagagacaaagtgcattga, and reverse 5′-tgggaaccccaaacttaaca; for *GATA3*, forward 5′-tgcctttggtttttagacagg, and reverse 5′-tcagcatcagaattccattcc. Quantitative PCR (qPCR) was carried out using LightCycler® 480 SYBR Green I Master (Roche) in the LightCycler® 480 system (Roche). All primer pairs were optimized to ensure specific amplification of the PCR product and the absence of any primer dimer. qPCR standard curves were set up for all. Quantification was calculated using the comparative *C*t method. Relative quantification (DCt) was performed using *GATA3* as reference gene. Each qPCR experiment contained triplicates of control or patient samples for all primer pairs.

### Statistical analyzes

Statistical significance was assessed using Mann-Whitney test and Student t-test for the comparison of DCt mean values between patients and controls or between carrier and non-carrier of the disease haplotype in pedigree A. For the analysis of DCt values, all possible combinations of nine affected individuals taken from their corresponding nine families were compared to controls, as a method of resampling. A hierarchical cluster analysis was used to identify clusters of individuals taking into consideration the two DCt values corresponding to the ICCA.SRa and ICCA.SRb subregions. Fisher's exact test was used to compare the frequencies between patients and controls in Cluster1.

## Results and Discussion

### Whole genome CGH analysis suggests higher frequency of low copy number of Variation_7105 in ICCA families

**Figure 1:**
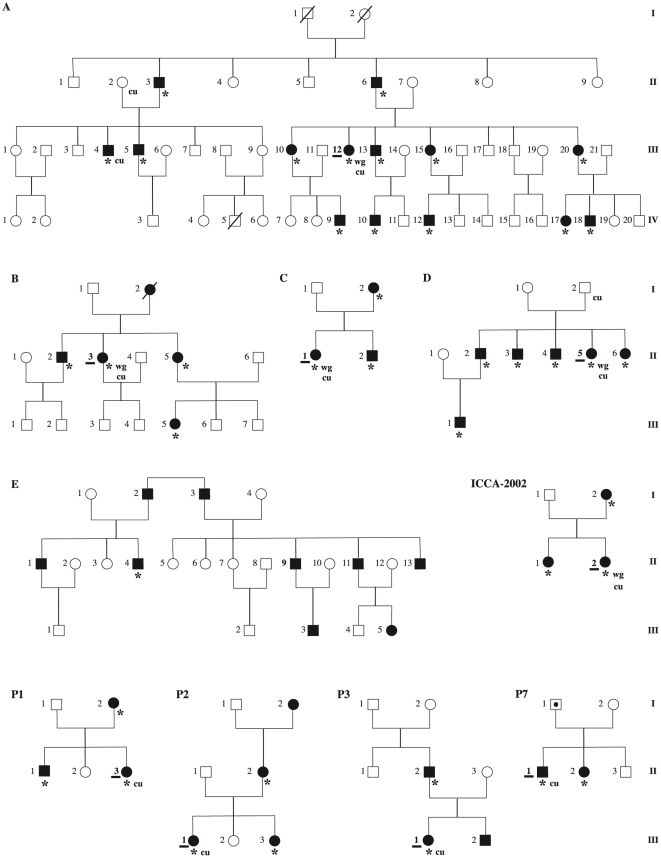
Pedigrees of the ten ICCA families. All pedigrees have already been reported: A to D[2], E[3], ICCA-2002[13], P1 to P3 and P7[33]. Pedigree A is also depicted in Figure S2. Black squares/circles: patients with ICCA syndrome (benign infantile seizures and/or paroxysmal dyskinesia). Empty squares/circles: unaffected individuals. Dotted square: phenotype unknown. Asterisks indicate the patients with DNAs available. Patients with underlined IDs correspond to those that were randomly selected for the first series (series S1) of qPCR experiments and that were used for hierarchical cluster analysis of qPCR data (Figure 3, Figure S2). wg: corresponds to the patients used for whole genome CGH (comparative genome hybridization) array studies. cu: corresponds to the patients and to the non-affected individuals used for custom CGH array studies.

**Figure 2:**
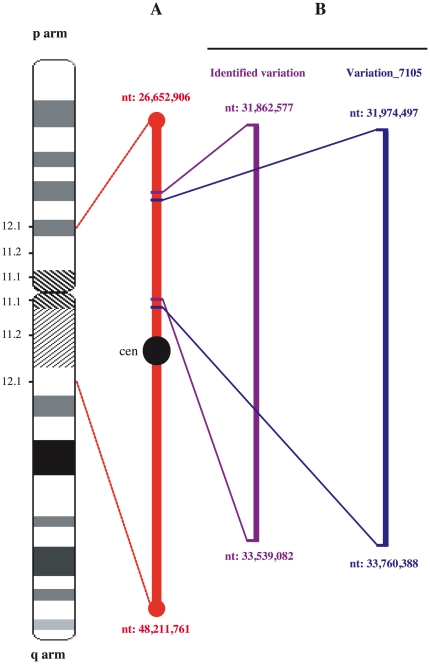
Schematic map of the pericentromeric region of human chromosome 16. (A) ICCA consensus region[5]. cen: centromere. nt: nucleotides. (B) Location of the copy number variation (CNV) in the ICCA patients (‘Identified variation’) and of the previously reported CNV (Variation_7105)[23]. Physical locations are indicated according to the NCBI Build 36.1 version (hg18) of the human genome sequence assembly at University of California Santa Cruz website (http:genome.ucsc.edu/). The most updated version (hg19) of the human genome sequence assembly has different nt positions with respect to hg18: for instance, hg18 position 31,862,577 corresponds to 31,955,076 in hg19, and hg18 position 33,539,082 to position 33,631,581 in hg19.

As a first attempt to test for the possible relationship of the ICCA syndrome with genomic events, one patient was randomly taken from each of five ICCA families from Northern France previously reported, i.e. pedigrees A to D[2] plus one additional pedigree thereafter designated as ICCA-2002[13] (Figure 1). Whole genome CGH analysis using Agilent 244K high density microarray revealed the existence of low copy number of a 1.7 megabases (Mb) genomic area at chromosome 16p11.2 (Figure 2) in four out of the five ICCA patients (Figure S1). In those four patients, the genomic region extended from 31,862,577 nucleotide (nt) position on its telomeric side, to 33,539,082 nt on its centromeric side (positions according to the hg18 human reference sequence NCBI Build 36.1 at UCSC website: http://genome.ucsc.edu/). This corresponded roughly to a copy number variation (CNV) known as Variation_7105 (from 31,974,497 nt to 33,760,388 nt, NCBI Build 36.1, according to the database of genomic variants at http://projects.tcag.ca/variation/). Variation_7105 has been identified previously in eight out of 50 control individuals who also originated from Northern France and using Agilent 185K and custom microarrays[23]. In the five patients studied here, no other genomic alteration was consistently detected in the ICCA area. In particular, the more telomeric deletion/duplication events reported at 16p11.2 in other patients with various phenotypes such as autism, schizophrenia, speech/language delay, obesity, or epileptic seizures[24]–[29] were not found. Particularly, the *de novo* 544-kb deletion at 16p11.2 (nt 29,560,500 to 30,104,842) that was recently reported in one atypical patient with kinesigenic PD (PKD) and with other clinical features including verbal learning disability and DOPA-responsive parkinsonism, and with possible infantile-onset convulsions[34], was not detected in any of the five and unrelated ICCA patients studied here. The tendency (4/5 patients here, as compared with 8/50 controls in previous study) towards a possible increase in the frequency of low copy number of Variation_7105 located right within the critical ICCA area, that in turn had previously and independently shown linkage with the ICCA syndrome, led us to further investigate in the patients the ICCA region at the genomic level.

### Quantitative PCR confirms a shift towards low copy number of Variation_7105 in the ICCA families

**Figure 3:**
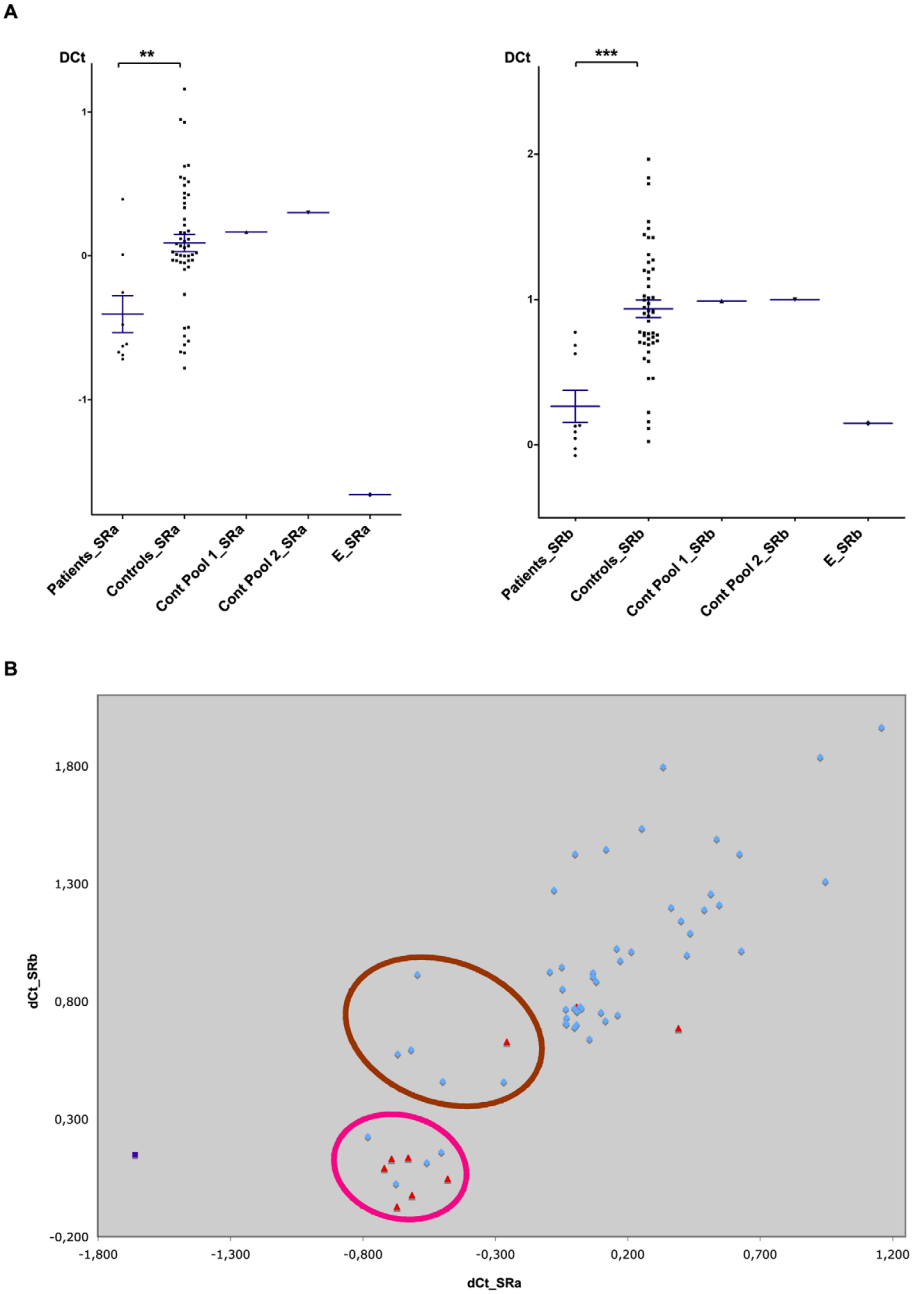
Quantitative PCR studies. Two specific subregions (ICCA.SRa and ICCA.SRb) of Variation_7105 were analyzed in nine unrelated ICCA patients (series S1) and in unrelated control individuals. (A) Comparisons of DCt values for ICCA.SRa (left) and ICCA.SRb (right) between ICCA patients and controls. (**) indicates P<0.01. (***) indicates P<0.001. DCt values of the control DNA pool (Promega) used for whole genome CGH array experiments (Cont Pool 1), of the pool of 50 DNAs of control individuals (Cont Pool 2), and of patient II.4 from pedigree E of Chinese origin[3], are also indicated. (B) Clustering analysis of DCt values for ICCA.SRa and ICCA.Srb in ICCA patients (red triangles) and controls (blue dots). Patient II.4 of pedigree E[3] is also indicated (purple square). The two clusters (Cluster 1, pink circle; Cluster 2, brown circle) that could be statistically isolated from all other values are indicated (see also Figure S2).

In order to confirm the data obtained by whole genome CGH arrays and to extend the analysis to more ICCA patients and to control individuals, quantitative polymerase chain reaction (qPCR) experiments were performed. Primers for qPCR were selected in two specific subregions (thereafter named ICCA.SRa and ICCA.SRb, respectively) of Variation_7105 that appeared unique as determined by *in silico* analysis based on the UCSC human genome assembly (NCBI Build 36.1). qPCR was performed on the five ICCA patients previously used for whole genome CGH array analyzes as well as on four patients from additional ICCA families (pedigrees P1, P2, P3 and P7 in Figure 1) also of European descent [33]. In each of the nine ICCA families, one patient was randomly chosen for the analysis (Figure 1). In parallel, 50 unrelated and healthy individuals (25 males and 25 females) of European origin were also analyzed (control individuals). The qPCR data clearly showed a shift towards low copy number in each of the ICCA.SRa and ICCA.SRb subregions in the nine ICCA patients (thereafter designated as series S1) as compared with the 50 control individuals (Figure 3A). The difference between ICCA patients and control individuals was statistically highly significant (p = 0.0036 for ICCA.SRa and p = 0.0001 for ICCA.SRb, Mann-Whitney test; p = 0.0049 for ICCA.SRa and p = 0.0001 for ICCA.SRb, unpaired t-test with Welch's correction). One additional patient was randomly taken from a family of Chinese origin (patient II.4 in pedigree E)[3] (Figure 1) that had shown significant linkage to the ICCA region. Although not taken into account for the statistics because of different ethnic origin of pedigree E, this patient yielded amongst the lowest DCt values (Figure 3). When the qPCR data were analyzed in the control individuals, no significant difference was found between males and females (p = 0.6671, Mann Whitney test, and p = 0.9757, unpaired t-test, for marker ICCA.SRa; p = 0.4879, Mann Whitney test, and p = 0.4389, unpaired t-test, for marker ICCA.SRb); hence the significant data depicted above were not due to any kind of gender bias. Importantly, the DNA pool (Cont Pool 1) that had been used as control DNA in the previous whole genome CGH array experiments showed DCt values in the same range as those obtained when considering the mean of the 50 control individuals DNAs mentioned above and as the mean DCt value obtained in another independent DNA pool (Cont Pool 2) of 50 novel healthy individuals (Figure 3A); this confirmed that the low copy numbers as detected by the CGH array analyses, had not been found because of a relative high copy number in the control DNA pool (Cont Pool 1) used for CGH.

The nine affected individuals used in the qPCR studies (series S1) and in the statistical analyses depicted just above were taken from nine corresponding ICCA families by random selection prior to the analysis; however, biased intrafamilial selection towards patients with low copy numbers could have occurred by chance. To address this issue and as the data were highly significant and comparable for both qPCR markers, all available DNAs from the other affected patients in each ICCA family were analyzed by qPCR for one of the two markers (marker ICCA.SRb) and the statistical analyses were redone for all possible combinations (30,240 in total) of nine patients (one per family). All resamplings of patients differed significantly from controls (p = 0.005 with a 95% confidence interval of 0.0049 to 0.0051).

### Clustering and intrafamilial qPCR analyzes

While genetic variation is inherently discrete at the nucleotide level, qPCR measurements are less discretely distributed because of measurement noise[35]. This indeed was oberved here when considering the two qPCR markers (corresponding to ICCA.SRa and ICCA.SRb, respectively) independendtly (Figure 3A). In contrast, a subset of the DCt values obtained in the nine ICCA patients of series S1 clustered into discrete groups when the two markers were considered together (Figure 3B; Figure S2). This was done by using a hierarchical cluster analysis in order to identify clusters of individuals, and by taking into consideration the two DCt values corresponding to the ICCA.SRa and ICCA.SRb subregions, respectively. Two clusters corresponding to individuals with low DCt values, and hence to individuals with low copy number of Variation_7105 (Clusters 1 & 2 in Figure 3B), could indeed be statistically isolated from all other values. In particular, Cluster 1 corresponded to the lowest combined DCt values: it comprised six out of the nine ICCA patients, but only four out of 50 control individuals (p = 0.0003, Fisher's exact test).

Overall, the qPCR data confirmed that ICCA patients have low copy number of the genomic area corresponding to Variation_7105 with higher frequency than control individuals. This was further confirmed by intrafamilial analysis. As pedigree A[2] was by far the largest ICCA pedigree ever described, we also performed the statistical analysis for DCt values of subregion ICCA.SRb within this family, by comparing all individuals of pedigree A who carried the disease haplotype with those who did not carry the disease haplotype. DCt values in haplotype carriers were lower than the DCt values of non carrier individuals and this difference was statistically significant (Mann-Whitney test, p = 0.0307; unpaired t-test, p = 0.0337) (Figure S3).

### Dedicated CGH array excludes a direct causal role for low copy number of Variation_7105

As mentioned above, the genomic area of low copy number in the ICCA families corresponded to the previously described Variation_7105 (http://projects.tcag.ca/variation/)[23]. However, the possibility remained, that slight differences in the genomic variant existed between ICCA patients and control individuals and hence that the genomic alteration in the patients was directly causing the phenotype. To address this issue, a more precise CGH analysis was performed by designing a chromosome 16p12.1-q12.1 dedicated 4×44 custom Agilent microarray enriched with 12,305 oligonucleotides between 27.5 Mb and 45.5 Mb. The novel microarray data confirmed and extended the whole genome CGH array data and the qPCR experiments depicted above in all nine ICCA patients of series S1 that were re-analyzed (Figure S4); moreover and as already indicated by our whole genome CGH array analysis, no copy number variation of the more telomeric region deleted in one peculiar patient with PKD and possibly infantile seizures[34], was found in any of the ICCA patients analyzed here with the chromosome 16p12.1-q12.1 enriched microarray. Genomic variant corresponding to Variation_7105 looked identical in size and overall copy number (Figure S4, panel A) between the six ICCA patients and the unaffected control individual (n°11) who all corresponded to Cluster 1-DNAs according to the qPCR experiments depicted above (Figure 3B, Figure S2). Similar results (Figure S4, panel B) were obtained when comparing DNAs of Cluster 2 corresponding to patient II.1 in pedigree C with DNAs from control individuals n°17 and 18. To further confirm the absence of any difference in the genomic region corresponding to Variation_7105 between patients and control individuals, direct competitive hybridization of patient II.5 in pedigree D versus control n°11, both belonging to Cluster 1 (Figure 3B, Figure S2), was performed (Figure S4, panel C); as expected, this experiment did not reveal any significant difference. Intrafamilial comparison between affected individual III.4 in pedigree A (haplotype carrier) and his unaffected and non-haplotype carrier mother (II.2) (Figure 1, Figure S3), and who displayed similar DCt values (Figure S3), was also done by direct competitive hybridization: again, there was no difference in the size and the copy number of Variation_7105 (Figure S4, panel D).

### The disease ICCA genetic defect lies close to or within Variation_7105

Altogether, these results confirmed that the genomic variant (Variation_7105) detected in the ICCA patients, although much more frequent than in control individuals, was very unlikely to directly cause the phenotype as it was also detected with no apparent difference in control individuals. Rather, the higher frequency of low copy number of Variation_7105 in the ICCA patients likely corresponded to linkage disequilibrium (LD) between Variation_7105 and the as yet unknown ICCA mutant allele. In the present case, the genetic defect causing ICCA could then be situated in the vicinity of Variation_7105 boundaries. Generally, the existence and complexity of LD between a given CNV and its neighboring single nucleotide variations (SNPs) has already been discussed[36]. Moreover, physiological inversions that would make it the picture more complicated have already been described in control individuals at chromosome 16p11[37]. The disease gene could also map right within Variation_7105. The corresponding genomic area is very gene-poor (http://genome.ucsc.edu/). It contains at least four nearly-identical copies of the *TP53TG3* gene encoding a tumor suppressor p53-inducible protein[38]. *TP53TG3* had been excluded by mutation screening in a previous study[39] but the detection of one mutant allele amongst several copies of the wild type allele could have been problematic. Interestingly, Variation_7105 also contains several highly homologous copies of the *SLC6A10P* pseudogene (Genbank NR_003083) corresponding to the family of creatine transporters; *SLC6A10P* sequences are indeed paralogous to the X-linked *SLC6A8* gene (also known as the *CT1* creatine transporter)[40] that is involved in various brain disorders including mental retardation and epileptic seizures[41]. Although *SLC6A10P* does not contain any open reading frame corresponding to a full-length creatine transporter[41], [42], the relationship with *SLC6A8* may not be coincidental by chance and warrants further investigations. As a matter of fact, *SLC6A10P* transcripts have been detected in the human fetal and adult brain[43]. In addition to *TP53TG3* and *SLC6A10P*, the existence of other genes or of sequences for small non-coding RNAs not yet detected in this complicated and pericentromeric area, cannot be excluded.

### Conclusion

We have shown here that low copy number of a 16p11 genomic variation (Variation_7105) situated in the critical region where the ICCA gene maps, is significantly much more frequent in ICCA patients than in control individuals. The other 16p11.2 disease-associated variations that had been detected in autism, in shizophrenia, in speech/language delay, in obesity, in epileptic seizures[24]–[30] or even in paroxysmal dyskinesia[34], were not detected in any of the patients tested here. Altogether, these data should now be taken into account in future studies that aim at identifying the genetic defect underlying the ICCA syndrome, by focusing on the 16p11 genomic area corresponding to Variation_7105 and to its surrounding regions. As compared with previously published data that led to define a critical ICCA region more than 20 Megabases large [5], narrowing down the critical region to Variation_7105 and to its immediate vicinity, clearly represents a significant progress.

## Supporting Information

Figure S1 Data of the whole genome CGH (comparative genome hybridization) array analysis (244K array from Agilent®).
(0.45 MB PDF)

Figure S2 Dendrogram from hierarchical cluster analysis of nine ICCA patients and 50 controls (numbered from 11 to 60).
(0.15 MB PDF)

Figure S3 Quantitative PCR study of the ICCA.SRb subregion in the largest ICCA family (Pedigree A).
(0.08 MB PDF)

Figure S4 Data of the chromosome 16p12.1-q12.1 dedicated CGH arrays (custom 4×44K array from Agilent®).
(0.31 MB PDF)

## References

[1] Caraballo R, Fejerman N, Caraballo R (2007). Benign familial and non familial infantile seizures.. Benign focal epilepsies in infancy, childhood and adolescence.

[2] Szepetowski P, Rochette J, Berquin P, Piussan C, Lathrop GM (1997). Familial infantile convulsions and paroxysmal choreoathetosis: a new neurological syndrome linked to the pericentromeric region of human chromosome 16.. Am J Hum Genet.

[3] Lee WL, Tay A, Ong, HT, Goh LM, Monaco AP (1998). Association of infantile convulsions with paroxysmal dyskinesias (ICCA syndrome): confirmation of linkage to human chromosome 16p12-q12 in a Chinese family.. Hum Genet.

[4] Szepetowski P, Fejerman N, Caraballo R (2007). Benign familial infantile seizures and paroxysmal choreoathetosis.. Benign focal epilepsies in infancy, childhood and adolescence.

[5] Rochette J, Roll P, Szepetowski P (2008). Genetics of infantile seizures with paroxysmal dyskinesia: the infantile convulsions and choreoathetosis (ICCA) and ICCA-related syndromes.. J Med Genet.

[6] Lee HY, Xu Y, Huang Y, Ahn AH, Auburger GW, Pandolfo M (2004). The gene for paroxysmal non-kinesigenic dyskinesia encodes an enzyme in a stress response pathway.. Hum Mol Genet.

[7] Rainier S, Thomas D, Tokarz D, Ming L, Bui M (2004). Myofibrillogenesis regulator 1 gene mutations cause paroxysmal dystonic choreoathetosis.. Arch Neurol.

[8] Weber YG, Storch A, Wuttke TV, Brockmann K, Kempfle J (2008). GLUT1 mutations are a cause of paroxysmal exertion-induced dyskinesias and induce hemolytic anemia by a cation leak.. J Clin Invest.

[9] Sadamatsu M, Masui A, Sakai T, Kunugi H, Nanko S (1999). Familial paroxysmal kinesigenic choreoathetosis: an electrophysiologic and genotypic analysis.. Epilepsia.

[10] Tomita H, Nagamitsu S, Wakui K, Fukushima Y, Yamada K (1999). Paroxysmal kinesigenic choreoathetosis locus map to chromosome 16p11.2-q12.1.. Am J Hum Genet.

[11] Swoboda KJ, Soong B, McKenna C, Brunt ER, Litt M (2000). Paroxysmal kinesigenic dyskinesia and infantile convulsions: clinical and linkage studies.. Neurology.

[12] Bennett LB, Roach ES, Bowcock AM (2000). A locus for paroxysmal kinesigenic dyskinesia maps to human chromosome 16.. Neurology.

[13] Thiriaux A, de St Martin A, Vercueil L, Battaglia F, Armspach JP (2002). Co-occurrence of infantile epileptic seizures and childhood paroxysmal choreoathetosis in one family: clinical, EEG, and SPECT characterization of episodic events.. Mov Disord.

[14] Striano P, Lispi ML, Gennaro E, Madia F, Traverso M (2006). Linkage analysis and disease models in benign familial infantile seizures: a study of 16 families.. Epilepsia.

[15] Caraballo R, Pavek S, Lemainque A, Gastaldi M, Echenne B (2001). Linkage of benign familial infantile convulsions to chromosome 16p12-q12 suggests allelism to the infantile convulsions and choreoathetosis syndrome.. Am J Hum Genet.

[16] Weber YG, Berger A, Bebek N, Maier S, Karafyllakes S (2004). Benign familial infantile convulsions: linkage to chromosome 16p12-q12 in 14 families.. Epilepsia.

[17] Callenbach PM, van den Boogerd EH, de Coo RF, ten Houten R, Oosterwijk JC (2005). Refinement of the chromosome 16 locus for benign familial infantile convulsions.. Clin Genet.

[18] Loftus BJ, Kim UJ, Sneddon VP, Kalush F, Brandon R (1999). Genome duplications and other features in 12 Mb of DNA sequence from human chromosome 16p and 16q.. Genomics.

[19] Martin J, Han C, Gordon LA, Terry A, Prabhakar S (2004). The sequence and analysis of duplication-rich human chromosome 16.. Nature.

[20] Iafrate AJ, Feuk L, Rivera MN, Listewnik ML, Donahoe PK (2004). Detection of large-scale variation in the human genome.. Nat Genet.

[21] Sharp AJ, Locke DP, McGrath SD, Cheng Z, Bailey JA (2005). Segmental duplications and copy-number variation in the human genome.. Am J Hum Genet.

[22] Redon R, Ishikawa S, Fitch KR, Feuk L, Perry GH (2006). Global variation in copy number in the human genome.. Nature.

[23] de Smith AJ, Tsalenko A, Sampas N, Scheffer A, Yamada NA (2007). Array CGH analysis of copy number variation identifies 1284 new genes variant in healthy white males: implications for association studies of complex diseases.. Hum Mol Genet.

[24] Ballif BC, Hornor SA, Jenkins E, Madan-Khetarpal S, Surti U (2007). Discovery of a previously unrecognized microdeletion syndrome of 16p11.2-p12.2.. Nat Genet.

[25] Kumar RA, KaraMohamed S, Sudi J, Conrad DF, Brune C (2008). Recurrent 16p11.2 microdeletions in autism.. Hum Mol Genet.

[26] Weiss LA, Shen Y, Korn JM, Arking DE, Miller DT (2008). Association between microdeletion and microduplication at 16p11.2 and autism.. N Engl J Med.

[27] Bochukova EG, Huang N, Keogh J, Henning E, Purmann C (2010). Large, rare chromosomal deletions associated with severe early-onset obesity.. Nature.

[28] Walters RG, Jacquemont S, Valsesia A, de Smith AJ, Martinet D (2010). A new highly penetrant form of obesity due to deletions on chromosome 16p11.2.. Nature.

[29] McCarthy SE, Makarov V, Kirov G, Addington AM, McClellan J (2009). Microduplications of 16p11.2 are associated with schizophrenia.. Nat Genet.

[30] Shinawi M, Liu P, Kang SH, Shen J, Belmont JW (2010). Recurrent reciprocal 16p11.2 rearrangements associated with global developmental delay, behavioral problems, dysmorphism, epilepsy, and abnormal head size.. J Med Genet.

[31] Lee JA, Lupski JR (2006). Genomic rearrangements and gene copy-number alterations as a cause of nervous system disorders.. Neuron.

[32] Lupski JR (2007). Genomic rearrangements and sporadic disease.. Nat Genet.

[33] Rochette J, Roll P, Fu YH, Lemoing AG, Royer-Zemmour B (2010). Novel familial cases with ICCA (infantile seizures with paroxysmal dyskinesia) syndrome.. Epileptic Dis.

[34] Lipton J, Rivkin MJ (2009). 16p11.2-related paroxysmal kinesigenic dyskinesia and dopa-responsive parkinsonism in a child.. Neurology.

[35] Locke DP, Sharp AJ, McCarroll SA, McGrath SD, Newman TL (2006). Linkage disequilibrium and heritability of copy-number polymorphisms within duplicated regions of the human genome.. Am J Hum Genet.

[36] McCarroll SA, Altshuler DM (2007). Copy-number variation and association studies of human disease.. Nat Genet.

[37] Kidd JM, Cooper GM, Donahue WF, Hayden HS, Sampas N (2008). Mapping and sequencing of structural variation from eight human genomes.. Nature.

[38] Ng CC, Koyama K, Okamura S, Kondoh H, Takei Y (1999). Isolation and characterization of a novel TP53-inducible gene, TP53TG3.. Genes Chromosomes Cancer.

[39] Kikuchi T, Nomura M, Tomita H, Harada N, Kanai K (2007). Paroxysmal kinesigenic choreoathetosis (PKC): confirmation of linkage to 16p11-q21, but unsuccessful detection of mutations among 157 genes at the PKC-critical region in seven PKC families.. J Hum Genet.

[40] Eichler EE, Lu F, Shen Y, Antonacci R, Jurecic V (1996). Duplication of a gene-rich cluster between 16p11.1 and Xq28: a novel pericentromeric-directed mechanism for paralogous genome evolution.. Hum Mol Genet.

[41] Hahn KA, Salomons GS, Tackels-Horne D, Wood TC, Taylor HA (2002). X-linked mental retardation with seizures and carrier manifestations is caused by a mutation in the creatine-transporter gene (SLC6A8) located in Xq28.. Am J Hum Genet.

[42] Höglund PJ, Adzic D, Scicluna SJ, Lindblom J, Fredriksson R (2005). The repertoire of solute carriers of family 6: identification of new human and rodent genes.. Biochem Biophys Res Commun.

[43] Bayou N, M'rad R, Belhaj A, Daoud H, Zemni R (2008). The creatine transporter gene paralogous at 16p11.2 is expressed in human brain.. Comp Funct Genomics,.

