# Supplementary material for: Infantile Convulsions with Paroxysmal Dyskinesia (ICCA Syndrome) and Copy Number Variation at Human Chromosome 16p11
Source: PLoS One. 2010 Oct 29;5(10):e13750. doi: 10.1371/journal.pone.0013750 (PMC2966418; doi:10.1371/journal.pone.0013750)
Supplement: Figure S1 — Data of the whole genome CGH (comparative genome hybridization) array analysis (244K array from Agilent®). (0.45 MB PDF) [file pone.0013750.s001.pdf]

Figure S1. Data of the whole genome CGH (comparative genome hybridization) array analysis (244K array from Agilent®). Array CGH profiles shown here focus on the pericentromeric region of human chromosome 16, from 27 Mb to 36 Mb. Squared areas correspond to CNV_7105 [23] (chr16:31,974,497 to 33,760,388; database of genomic variants, hg18, at http://projects.tcag.ca/variation/). In each panel, longitudinal axis represents the position of probes located in genome and horizontal axis represents the log2 ratio test/control of the probes. Each dot represents a single probe spotted on the array. Dots with a value of zero represent equal fluorescence intensity ratio between sample and control.

(A)Hybridization of patient DNA (III.12, pedigree A) versus Promega reference DNA.

(B) Hybridization of patient DNA (II.3, pedigree B) versus Promega reference DNA.

(C) Hybridization of patient DNA (II.1, pedigree C) versus Promega reference DNA.

(D) Hybridization of patient DNA (II.5, pedigree D) versus Promega reference DNA.

(ICCA-2002) Hybridization of patient DNA (II.2, pedigree ICCA-2002) versus Promega reference DNA.

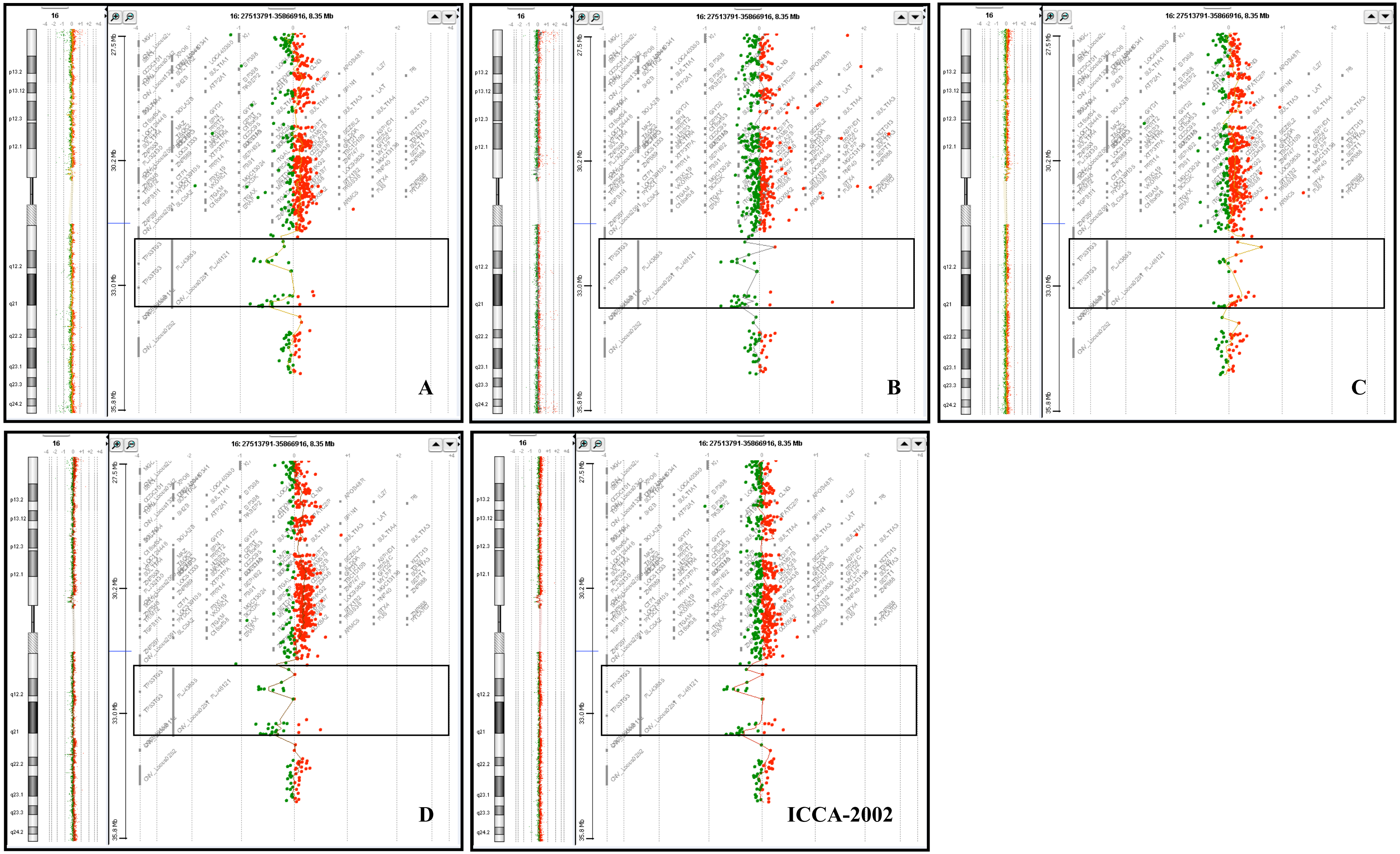
16: 27513791-35866916, 8.35 Mb
A
16: 27513791-35866916, 8.35 Mb
B
16: 27513791-35866916, 8.35 Mb
C
16: 27513791-35866916, 8.35 Mb
D
16: 27513791-35866916, 8.35 Mb
ICCA-2002
