# Supplementary material for: Infantile Convulsions with Paroxysmal Dyskinesia (ICCA Syndrome) and Copy Number Variation at Human Chromosome 16p11
Source: PLoS One. 2010 Oct 29;5(10):e13750. doi: 10.1371/journal.pone.0013750 (PMC2966418; doi:10.1371/journal.pone.0013750)
Supplement: Figure S2 — Dendrogram from hierarchical cluster analysis of nine ICCA patients and 50 controls (numbered from 11 to 60). (0.15 MB PDF) [file pone.0013750.s002.pdf]

Figure S2. Dendrogram from hierarchical cluster analysis of nine ICCA patients and 50 controls (numbered from n°11 to n°60). All nine ICCA patients studied are indicated as follows: pedigree symbol/individual ID. Two clusters (Cluster 1 and Cluster 2, boxed) could be statistically separated from each other and from all other Dct values ($p<0.0001$ for ICCA.SRa and ICCA.SRb, Kruskal Wallis test).

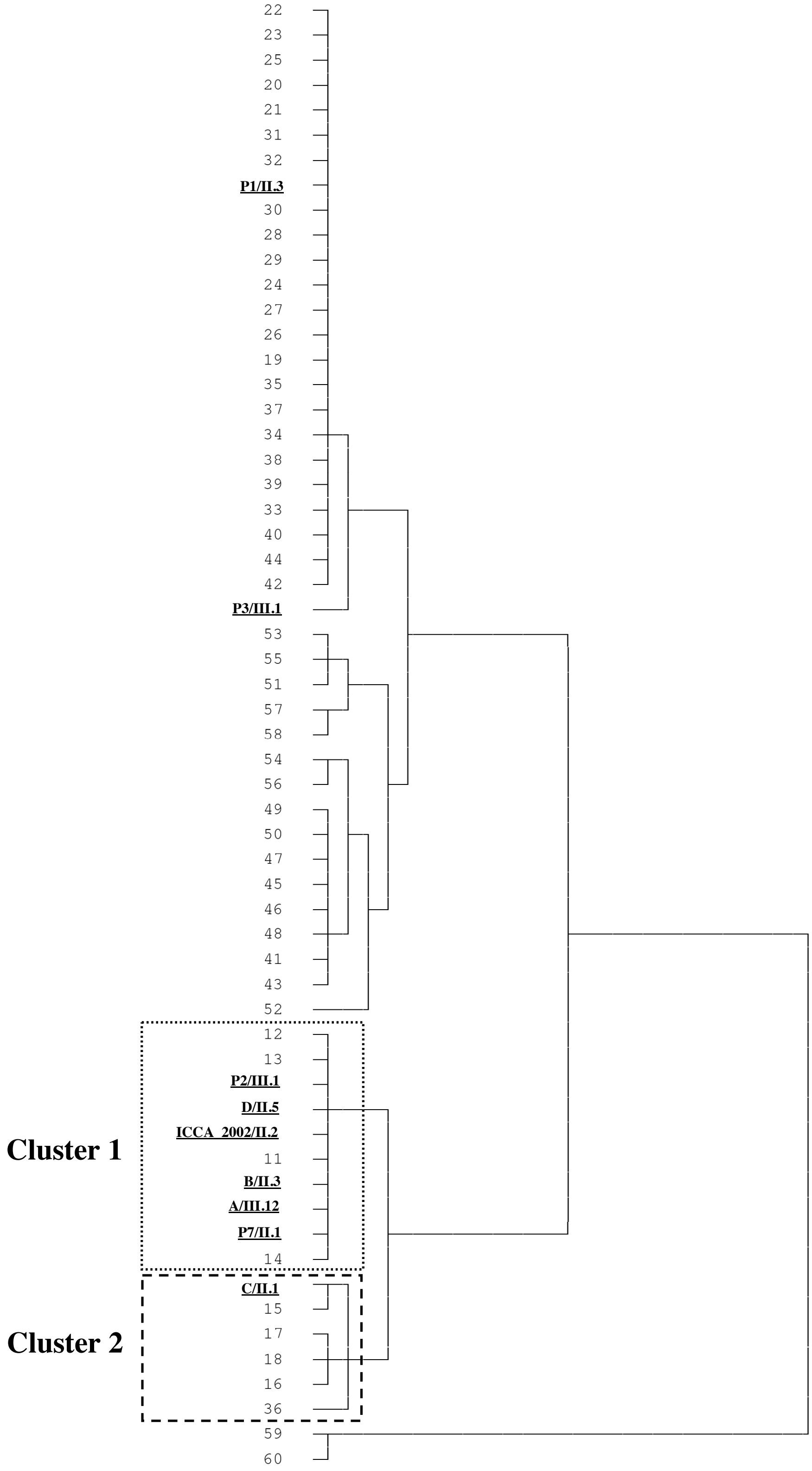
22
23
25
20
21
31
32
P1/II.3
30
28
29
24
27
26
19
35
37
34
38
39
33
40
44
42
P3/III.1
53
55
51
57
58
54
56
49
50
47
45
46
48
41
43
52
12
13
P2/III.1
D/II.5
ICCA 2002/II.2
11
B/II.3
A/III.12
P7/II.1
14
C/II.1
15
17
18
16
36
59
60
Cluster 1
Cluster 2
