# Supplementary material for: Infantile Convulsions with Paroxysmal Dyskinesia (ICCA Syndrome) and Copy Number Variation at Human Chromosome 16p11
Source: PLoS One. 2010 Oct 29;5(10):e13750. doi: 10.1371/journal.pone.0013750 (PMC2966418; doi:10.1371/journal.pone.0013750)

Figure S3. Quantitative PCR study of the ICCA.SRb subregion in the largest ICCA family (Pedigree A).[2] DCt values are indicated for each individual carrying (asterisk) or not the disease haplotype. Black circles and squares indicate females and males affected with benign infantile convulsions (left-filled), paroxysmal dyskinesia (right-filled), or both (fully filled). Empty circles and squares indicate unaffected females and males, respectively.

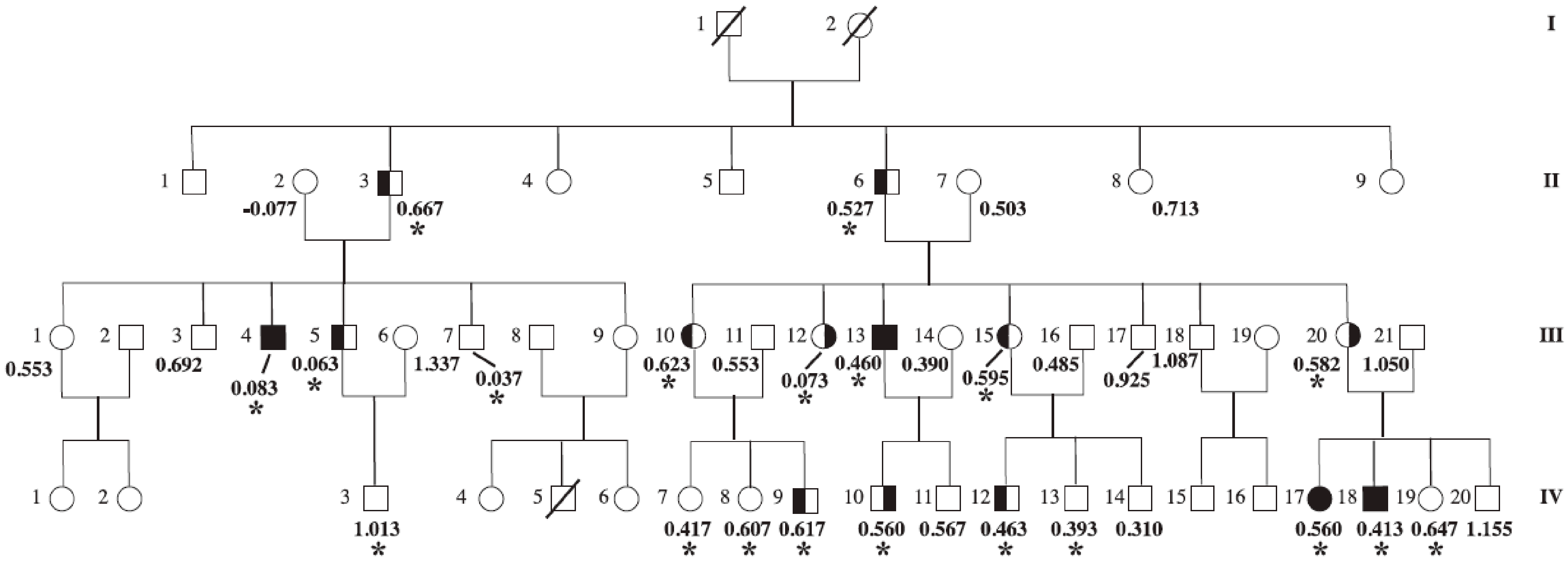

Supplement: Figure S3 — Quantitative PCR study of the ICCA.SRb subregion in the largest ICCA family (Pedigree A). (0.08 MB PDF) [file pone.0013750.s003.pdf]
