# Supplementary material for: Infantile Convulsions with Paroxysmal Dyskinesia (ICCA Syndrome) and Copy Number Variation at Human Chromosome 16p11
Source: PLoS One. 2010 Oct 29;5(10):e13750. doi: 10.1371/journal.pone.0013750 (PMC2966418; doi:10.1371/journal.pone.0013750)
Supplement: Figure S4 — Data of the chromosome 16p12.1-q12.1 dedicated CGH arrays (custom 4×44K array from Agilent®). (0.31 MB PDF) [file pone.0013750.s004.pdf]

**Figure S4.** Data of the chromosome 16p12.1-q12.1 dedicated CGH arrays (custom 4X44K array from Agilent®). Array CGH profiles shown here focus on the pericentromeric region of human chromosome 16, from 26-27 Mb to 35-36 Mb. Squared areas correspond to CNV_7105 [23] (chr16:31,974,497 to 33,760,388; database of genomic variants, hg 18, at http://projects.tcag.ca/variation/). In each panel, longitudinal axis represents the position of probes located in genome and horizontal axis represents the log2 ratio test/control of the probes.

(A) Hybridization of one patient DNA (II.1, pedigree P7) (red line) and of one unaffected individual (n°11) (blue line), both from Cluster 1 (Figure 3B, Figure S2), versus Promega reference DNA. Note that the two profiles show no detectable difference.

(B) Hybridization of one patient DNA (II.1, pedigree C) (red line) and of one unaffected individual (n°17) (blue line), both from Cluster 2 (Figure 3B, Figure S2), versus Promega reference DNA. Note that the two profiles show no detectable difference.

(C) Hybridization of one patient DNA (II.5, pedigree D) versus one unaffected individual (n°11), both from Cluster 1 (Figure 3B, Figure S2). No difference between the two DNAs is detected (blue line).

(D) 'Intrafamilial' hybridization of one patient DNA (III.4, pedigree A) versus his unaffected mother (II.2, pedigree A). No difference between the two DNAs is detected (blue line).

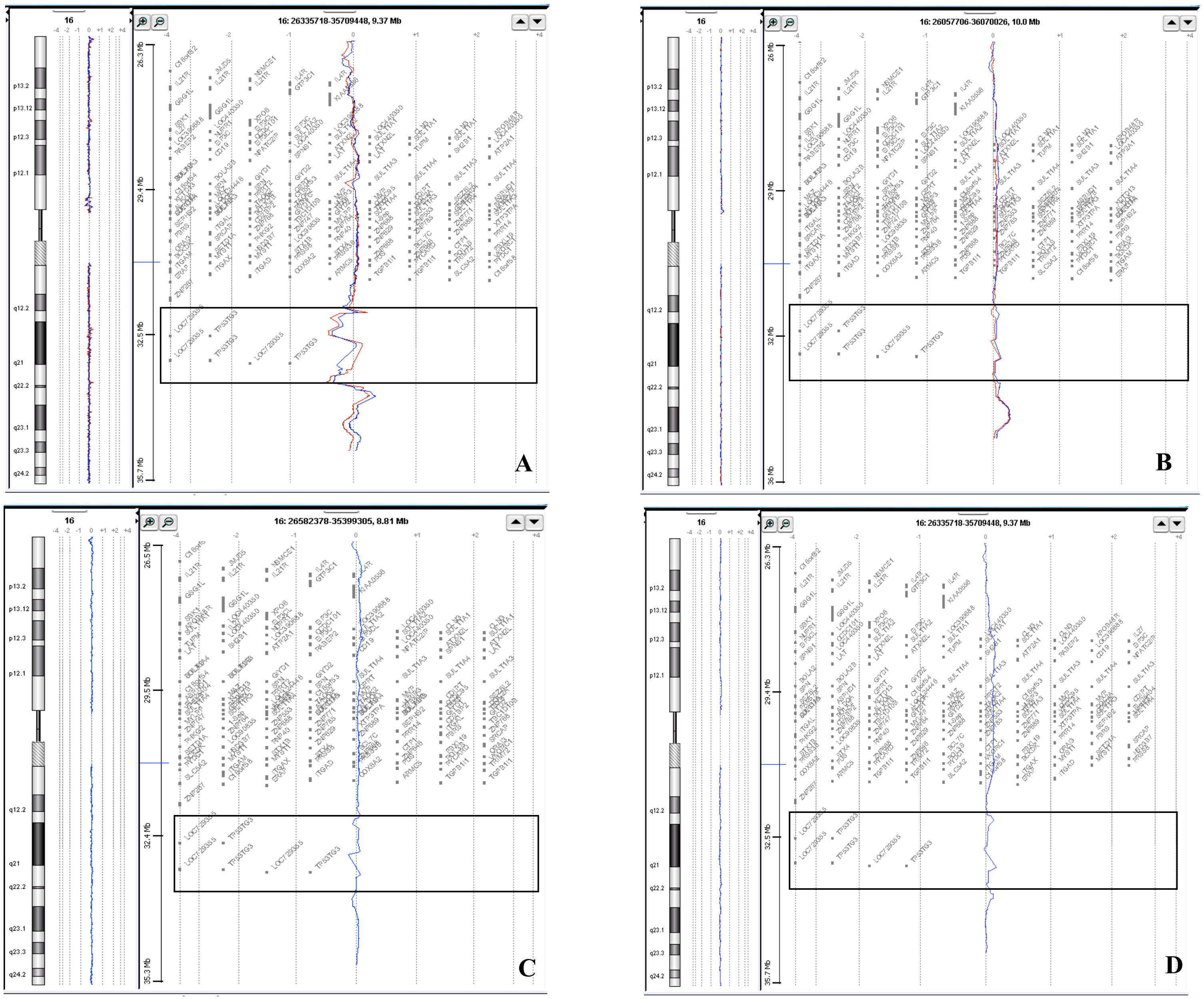
16: 26335718-35709448, 9.37 Mb
A
16: 26057706-36070026, 10.0 Mb
B
16: 26582378-35399305, 8.81 Mb
C
16: 26335718-35709448, 9.37 Mb
D
